# Health effects associated with foods characteristic of the Nordic diet: a systematic literature review

**DOI:** 10.3402/fnr.v57i0.22790

**Published:** 2013-10-09

**Authors:** Agneta Åkesson, Lene F. Andersen, Ása G. Kristjánsdóttir, Eva Roos, Ellen Trolle, Eeva Voutilainen, Elisabet Wirfält

**Affiliations:** 1 Nutritional Epidemiology Unit, Institute of Environmental Medicine, Karolinska Institutet, Stockholm, Sweden; 2 Department of Nutrition, Institute of Basic Medical Sciences, University of Oslo, Oslo, Norway; 3 Unit for Nutrition Research, University of Iceland, Reykjavík, Iceland; 4 Folkhälsan Research Center, Helsinki, Finland; 5 Department of Public Health, Hjelt Institute, University of Helsinki, Helsinki, Finland; 6 Division of Nutrition, National Food Institute, Technical University of Denmark, Søborg, Denmark; 7 Open University, University of Helsinki, Helsinki, Finland; 8 Department of Clinical Sciences in Malmö, Lund University, Lund, Sweden

**Keywords:** Nordic Nutrition Recommendations, systematic literature review, food-based dietary guidelines, berries, potatoes, whole grains, milk, meat

## Abstract

**Background:**

In preparing the fifth edition of the Nordic Nutrition Recommendations (NNR), the scientific basis of specific food-based dietary guidelines (FBDG) was evaluated.

**Objective:**

A systematic review (SR) was conducted to update the NNR evidence based on the association between the consumption of potatoes, berries, whole grains, milk and milk products, and red and processed meat, and the risk of major diet-related chronic diseases.

**Design:**

The SR was based on predefined research questions and eligibility criteria for independent duplicate study selection, data extraction, and assessment of methodological quality and applicability. We considered scientific data from prospective observational studies and intervention studies, published since year 2000, targeting the general adult population. Studies of meat and iron status included children, adolescents, and women of childbearing age.

**Results:**

Based on 7,282 abstracts, 57 studies met the quality criteria and were evidence graded. The data were too limited to draw any conclusions regarding: red and processed meat intake in relation to cardiovascular disease (CVD) and iron status; potatoes and berries regarding any study outcomes; and dairy consumption in relation to risk of breast cancer and CVD. However, dairy consumption seemed unlikely to increase CVD risk (moderate-grade evidence). There was probable evidence (moderate-grade) for whole grains protecting against type 2 diabetes and CVD, and suggestive evidence (low-grade) for colorectal cancer and for dairy consumption being associated with decreased risk of type 2 diabetes and increased risk of prostate cancer. The WCRF/AICR concludes that red and processed meat is a convincing cause of colorectal cancer.

**Conclusions:**

Probable (moderate) evidence was only observed for whole grains protecting against type 2 diabetes and CVD. We identified a clear need for high-quality nutritional epidemiological and intervention studies and for studies of foods of the Nordic diet.

The Nordic Nutrition Recommendations (NNR) aim to promote good overall health and to reduce the risk of diet-related chronic diseases common in Nordic populations (1). Previous editions focused mainly on setting nutrient reference values. In preparation of the fifth edition of the NNR, the scientific basis for specific common food-based dietary guidelines (FBDG) was evaluated.

Diets of the Nordic countries share some common foods and processing methods including boiled potatoes, berries, whole-grain wheat and rye bread, breakfast cereals, and fermented milk, which potentially confer specific health benefits. Regarding meat consumption, the dietary recommendations to prevent cancer issued by the World Cancer Research Fund/American Institute for Cancer Research (WCRF/AICR) advocate increased consumption of plant food at the expense of red and processed meat (2). These recommendations resemble the dietary guidelines of many Westernised countries, but because meat is considered an important source of iron (1), limiting red meat consumption may put certain population groups at higher risk of mineral (i.e. iron) deficiency. The influence of these food groups on certain conditions as well as on the development of chronic disease needs to be systematically reviewed.

This study aims to conduct a systematic review (SR) of the scientific evidence regarding the association between the consumption of five key food groups in the Nordic countries and the development of major diet-related chronic diseases, to provide a basis for FBDG. The SR was based on predefined research questions and eligibility criteria for independent duplicate study selection, data extraction, and assessment of methodological quality and applicability. We reviewed scientific data from prospective observational studies and randomised intervention studies targeting the general adult population, published between January 2000 and September 2010, exploring associations between five food groups commonly consumed in Nordic countries, and the risk of diseases or intermediate biomarkers of these diseases. Based on a three-category quality grading system, we summarised the evidence quality (3–5) for findings regarding each food group and outcome. An updated search was performed (February 2012) for three of the five food groups.

## Material and methods

### Research questions

#### Definition of food groups

The five food groups included and their respective definitions are as follows:

- Potatoes were defined as total potatoes; if possible, different forms of preparation were considered separately.
- Berries include all varieties and preparation methods except extracts or freeze-dried products.
- Whole grains, including wheat, rye, oats, and barley, were defined according to the definitions used in each study.
- Milk and milk products included all types of dairy products, except butter.
- Red meat was defined as beef, lamb, and pork; processed meat has no generally agreed-on definition, the term being inconsistently used in epidemiological studies. ‘Processed meat’ is defined in the WCRF/AICR report as meat (usually red meat) preserved by smoking, curing, or salting, or by the addition of preservatives. Meats preserved only by refrigeration or cooked meat are usually not classified as processed meat. Ham, bacon, pastrami, salami, sausages, bratwursts, frankfurters, and hot dogs are processed meat. Minced meats are regarded as processed meat if preservatives have been added (2).

#### Outcomes

The included outcomes considered relevant to Nordic populations are as follows:

- Cardiovascular disease, coronary heart disease (CVD/CHD) incidence and mortality, stroke (including various subtypes), heart failure, and venous thromboembolism are examples of specific diagnoses included;
- Type 2 diabetes, including related intermediate biomarkers of type 2 diabetes;
- Inflammatory factors: interleukin-6 (IL-6), its soluble receptor (sIL-6R), high-sensitivity C-reactive protein (hsCRP), and tumour necrosis factor-alpha (TNF-alpha);
- Colorectal, prostate, and breast cancer incidence and mortality;
- Bone health, defined as fracture incidence and changes in bone mineral density; and
- Iron status, defined based on serum ferritin and haemoglobin or haematocrit; the definitions used by individual studies of low or optimal iron status were applied.

#### Definition of research questions

The specific outcomes considered relevant to each food group are outlined, together with the research questions, in Tables 1 and 2.

**Table 1:** The research questions for prospective observational studies*

1.	What is the association between potato consumption and CVD/CHD incidence and mortality, type 2 diabetes incidence, and inflammatory factors in the general population?
2.	What is the association between berry consumption and CVD/CHD incidence and mortality, type 2 diabetes incidence, and inflammatory factors in the general population?
3.	What is the association between whole grain consumption and CVD/CHD and colorectal cancer incidence and mortality, type 2 diabetes incidence, and inflammatory factors in the general population?
4.	What is the association between milk and milk product consumption and CVD/CHD and prostate and breast cancer incidence and mortality, type 2 diabetes incidence, bone health, and inflammatory factors in the general population?
5.	What is the association between red and processed meat and CVD/CHD and colorectal cancer incidence and mortality and inflammatory factors in the general population as well as iron status in children, adolescents, and women of childbearing age?

* The eligibility criteria for prospective observational studies are presented in Table 3.

**Table 2:** The research questions for intervention studies*

1.	What is the effect of potato consumption on CVD/CHD incidence and mortality, type 2 diabetes incidence and intermediate biomarkers of diabetes, and inflammatory factors in the general population?
2.	What is the effect of berry consumption on CVD/CHD incidence and mortality, type 2 diabetes incidence and intermediate biomarkers of diabetes, and inflammatory factors in the general population?
3.	What is the effect of whole-grain consumption on CVD/CHD incidence and mortality, type 2 diabetes incidence and intermediate biomarkers of diabetes, and inflammatory factors in the general population?
4.	What is the effect of milk and milk product consumption on CVD/CHD incidence and mortality, type 2 diabetes incidence and intermediate biomarkers of diabetes, and inflammatory factors in the general population?
5.	What is the effect of red and processed meat consumption on iron status in children, adolescents, and women of childbearing age?

* The eligibility criteria for intervention studies are presented in Table 3.

### Eligibility criteria

The eligibility criteria for the included studies are shown in Table 3, but are explained in detail elsewhere (3, 5). The research questions targeted the general apparently healthy adult population, with the exception of the association between meat and iron status, where children, adolescents, and women of childbearing age were targeted.

**Table 3:** Eligibility criteria

Population	General healthy population, adults (20 years of age or older at start of study); children, adolescents, and women of childbearing age were included in studies of iron status
Exposure of interest	See research questions
Outcome	See research questions
Type of study	Observational studies: prospective cohort studies and prospective (nested) case-control studies Randomized controlled intervention studies
Number of participants	Observational studies: no criteria Intervention studies: minimum of 20 in each group
Dietary assessment methods	Food frequency questionnaire (FFQ), multiple 24-h recalls (four or more), multiple dietary records (four or more), dietary history methodology
Publication languages	English and Nordic
Publication type	Original papers
Time period for screened publications	January 2000–September 2010; updated search until February 2012 for potatoes, milk and milk products, and red and processed meat
Length of follow-up	Observational studies: Incidences of diseases and mortality, 4 years or more Biomarkers of inflammation and iron status, 6 months or more Intervention studies: Biomarkers of inflammation, about 1 month or more Iron status, 3 months or more Bone mineral density, 2 years or more Intermediate biomarkers of diabetes, 8 weeks or more
Compliance	More than 50%

### Search strategy

Based on the research questions, a search strategy was constructed by a librarian (Appendix A). The search was performed in the Medline and Embase databases. All articles published between January 2000 and September 2010 were considered. For the food group potatoes, milk, and red or processed meat, the search was updated in February 2012. Although a search was conducted for the research question on red and processed meat and colorectal cancer, we decided to base the conclusions in this SR on the Summary Colorectal Cancer Report of May 2011, part of the continuous update project (6, 7); accordingly, the papers identified in the search on meat and colorectal cancer were not included in this SR.

### Evaluation and selection of papers

The abstracts identified using each search strategy were screened. Based on the eligibility criteria (Table 3), potentially relevant articles selected via the screening procedure were ordered in full text and reviewed. Only observational studies using a prospective design (i.e. cohort and nested case-control studies) or randomised intervention studies were considered (3). All studies meeting the eligibility criteria were then quality assessed using the Quality Assessment Tools (3) and subsequently quality graded A, B, or C (3–5). Studies rated C were excluded from further evaluation and are listed, together with the excluded studies (i.e. not meeting the eligibility criteria), in Appendix B. Study selection, data extraction, and methodological quality and applicability assessment were conducted independently by two reviewers (authors), and disagreement on the final quality grade (A–C) was resolved by consensus. The two reviewers jointly constructed the evidence and summary tables for each outcome and food group and, based on the three-category quality grading system, summarised the grade of evidence as: 1) convincing (high), 2) probable (moderate), 3) limited – suggestive (low), and 4) limited – no conclusion (insufficient). The criteria for assigning evidence grades were modified in light of those issued by the WCRF/AICR in 2007 (3).

The search strategy was repeated in 2012 to include studies published between the end of the first search and February 2012. As the work on whole grains and berries had been finalised at that time, the studies identified in the updated search were scanned and reviewed for only three food groups, that is, potatoes, milk, and red or processed meat. Only one reviewer conducted the updated search, the aim of which was to identify any studies that might contradict the conclusions based on the first search.

## Results

The numbers of abstracts and full-text papers reviewed for quality assessment in the SR for each food group and outcome are presented in Table 4. In Table 5, the results of the SR are summarised for the studies graded A or B.

**Table 4:** Overview of database search results (January 2000–September 2010) and the included number of papers

Food group	Number of abstracts retrieved	Potentially meeting eligibility criteria	Selected for quality assessment	Included studies by outcome*
Potatoes	367	11	2	1 – type 2 diabetes
Berries	958	10	7	2 – CVD/CHD 5 – inflammatory markers
Whole grains	1,919	32	20	6 – CVD/CHD 6 – type 2 diabetes 2 – inflammatory markers 4 – colorectal cancer
Milk and milk products	2,379	88	45	6 – CVD/CHD (+ 2 SRs) 7 – type 2 diabetes, intermediate biomarkers of diabetes 1 – inflammatory markers 8 – prostate cancer 3 – breast cancer 1 – bone health
Red and processed meat	1,659**	93	13	4 – CVD/CHD 1 – iron status

* Includes studies graded A or B, but not C.

** Includes abstracts on meat and colorectal cancer.

**Table 5:** Summary of the individual results, the strength of evidence, and the conclusion of the SR*

Food group	Outcome	Effect	Quality of included studies	Grade of evidence (high, moderate, low, insufficient)	Conclusion
Potatoes	CVD/CHD	No studies	**-**	Insufficient	No conclusion on the association between potato consumption and risk of CVD/CHD
	Type 2 diabetes	*Halton et al., 2006* (8)*, US women*. 60–120-Item FFQ (updated five times after baseline). Potatoes RR 1.14 (1.02–1.26) French fries RR 1.21 (1.09–1.33) comparing highest and lowest quintiles, corresponding to 0.63 and 0.07 serv/d and 0.14 and 0 serv/d, respectively, for potatoes (one baked or 1 cup mashed/serving) and French fries (113 g/serving). RR 1.18 (1.03–1.35) per continuous 1 cup mashed or baked. Associations persisted in obese but not in lean women. B	B=1	Insufficient	No conclusion on the association between potato consumption and risk of type 2 diabetes
	Inflammatory factors	No studies		Insufficient	No conclusion on the association between potato consumption and inflammatory markers
Berries	CVD/CHD	*Mink et al., 2000* (9)*, US women.* 127-Item FFQ. Blueberries and strawberries 0 servings/week compared with >0 servings/week. Lower incidence of CHD (blueberries RR 0.81 [0.69–0.95], strawberries 0.84 [0.74–0.95]) and CVD (blueberries RR 0.85 [0.75–0.96], strawberries 0.82 [0.74–0.89]) when age and energy adjusted. Strawberries lower incidence of CVD (RR 0.91 (0.82–1.00) also in multivariate adjusted model (adjusted for age, energy intake, marital status, education, blood pressure, diabetes, BMI, waist-to-hip ratio, physical activity, smoking, and oestrogen use). B *Sesso et al., 2007* (10)*, US women.* 131-Item semi-quantitative FFQ. Strawberry intake was not associated with the risk of incident CVD. Strawberries ≥2 servings/week compared with none. Total CVD RR=1.27 (0.94–1.72). Adjusted for age, treatment, lifestyle factors, dietary factors, and total energy intake. B	B=2	Insufficient	No conclusion on the association between berry consumption and risk of CVD/CHD
	Type 2 diabetes	No studies		Insufficient	No conclusion on the association between berry consumption and risk of type 2 diabetes
	Inflammatory markers	*Larmo et al., 2008* (11)*, Finnish men and women.* Serum CRP decreased significantly (median change –0.059 mg/L) after 8-wk intervention (28 g/day sea buckthorn). B *Larmo et al., 2009* (12)*, Finnish men and women.* There was no correlation between changes of plasma flavonols and changes of serum CRP in the intervention group (28 g/day sea buckthorn). B *Basu et al., 2010* (13)*, US men and women.* 3-Day food records at three time points, i.e. baseline, week 4, and week 8. No effect of intervention (a daily dose of 50 g freeze-dried blueberries ∼350 g fresh blueberries) on inflammatory factors, CRP, adiponectin, IL-6, soluble intercellular adhesion molecule-1, and vascular adhesion molecule-1. B *Karlsen et al., 2010* (14)*, Norwegian men and women.* 7-Day registration of fluid intake before inclusion and during last week of intervention. Among adults at increased risk of CVD, supplementation with bilberry juice (330 mL/d diluted with tap water to 1 L) reduced plasma concentrations of several NF-κB-regulated inflammatory mediators, i.e. CRP, IL-6, IL-15, and monokine induced by INF-γ (MIG). Unexpected increase in plasma TNF-α. B *Lehtonen et al., 2010* (15)*, Finnish women.* 3-Day food records in the middle of the intervention. No effect of intervention (daily intake of 163 g of various berries) on plasma CRP, TNF-α, or ORAC. Plasma adiponectin increased in intervention group. B	B=5	Insufficient	No conclusion on the association between berry consumption and risk of inflammatory markers
Whole grains	CVD/CHD CAD, HF, Stroke	*Liu et al., 2000* (16)*, US women*. 126-Item semi-quantitative FFQ. Ischemic stroke RR 0.69 (0.50–0.98) comparing highest quintile (median 2.7 servings/day) with lowest (median 0.13 servings/day). Adjusted for age and known CVD risk factors including total energy intake. B *Liu et al., 2003* (17)*, US men*. Simple semi-quantitative FFQ. CVD specific mortality RR 0.80 (CI 0.66–0.97) when comparing ≥1 servings/day to rare whole-grain cereal consumption. Adjusted for age, lifestyle factors, BMI, history of type 2 diabetes, high cholesterol, hypertension, and use of multivitamins. B *Steffen et al., 2003* (18), *US men and women*. 66-Item semi-quantitative FFQ at baseline and 6 years later. Coronary artery disease incidence HR 0.72 (CI 0.53–0.97) comparing highest quintile (2.0–10.5 servings/day) with lowest quintile (0–0.2 servings/day). Ischemic stroke incidence HR 0.75 (0.46–1.22), comparing highest quintile (2.0–10.5 servings/day) with lowest quintile (0–0.2 servings/day). Adjusted for age and known CVD risk factors including energy intake. B *Jensen et al., 2004* (19)*, US men.* 131-Item semi-quantitative FFQ. CHD HR 0.82 (95% CI: 0.70–0.96) comparing highest quintile (median 42.4 g/day) with lowest quintile (median 3.5 g/day). Adjusted for age and known CVD risk factors, including energy intake. B *Sahyoun et al, 2006* (20), *US men and women.* 3-Day food record. CVD mortality. A significant inverse trend was observed between whole-grain consumption and mortality from CVD (*p=*0.04), RR 0.48 (95% CI: 0.25–0.96), when comparing highest quartile (median 2.90 servings/day) with lowest quartile (median 0.31 servings/day). Adjusted for demographic and lifestyle factors. B *Nettleton et al., 2008* (21), *US men and women*. 66-Item semi-quantitative FFQ. Heart failure risk was lower with greater whole-grain intake RR 0.93 (95% CI: 0.87–0.99) per 1 serving/day. Adjusted for age, energy, demographic factors, lifestyle, and prevalent disease. B	B=6	Moderate	Probable protective association between whole-grain consumption and risk of CVD/CHD For specific endpoints (e.g. stroke) the evidence is insufficient
	Type 2 diabetes	Prospective studies *Liu et al., 2000* (22)*, US women.* 126-Item semi-quantitative FFQ. Highest quintile (1.77–15.93 servings/day) compared with lowest quintile (0–0.26 servings/day) RR 0.73 (0.63–0.85) *p* <0.0001. Adjusted for age, lifestyle, and known type 2 diabetes risk factors including total energy intake. B *Meyer et al., 2000* (23)*, US women.* 127-Item FFQ. Highest quintile (median 20.5 servings/week) compared with lowest quintile (median 1 servings/week) RR 0.79 (0.65–0.96) *p=*0.0089. Adjusted for age and known risk factors for type 2 diabetes, including total energy intake. B *Fung et al., 2002* (24)*, US men.* 131-Item semi-quantitative FFQ. Highest quintile (3.2–21.5 servings/day) compared with lowest quintile (0.0–0.4 servings/day) RR 0.70 (0.57–0.85) *p*<0.0006. Adjusted for age and known risk factors for type 2 diabetes, including energy intake. B *De Munter et al., 2007* (25)*, US women.* Semi-quantitative FFQ used 1984, 1986, 1990, 1994, and 1998 for NHSI study and 1991, 1995, and 1999 for NHSII. NHSI study highest quintile (36.9 g/day) compared with lowest quintile (3.2 g/day) RR 0.75 (0.68–0.83); *p* <0.001, NHSII study highest quintile (45.6 g/day) compared with lowest quintile (5.5 g/day) RR 0.86 (0.72–1.02), fourth quintile (26.3 g/day) compared with lowest quintile (5.5 g/day) RR 0.74 (0.63–0.86), *p=*0.03. Adjusted for age and known risk factors for type 2 diabetes, including total energy intake. B *Montonen et al., 2003* (26)*, Finnish men and women*. Dietary history of previous year. Highest whole-grain quartile (238–1321 g/day) compared with lowest quartile (0–109 g/day) RR 0.65 (0.36–1.18), third quartile (163–237 g/day) compared with lowest quartile (0–109 g/day) RR 0.52 (0.31–0.88) *p=*0.02. Adjusted for age, sex, geographic area, lifestyle, and intakes of energy, fruit and berries, and vegetables. B Intervention study: glucose/insulin factors *Juntunen et al., 2003* (27)*, Finnish women.* 4-d food records and daily records of the intake of bread. Acute insulin response increased significantly (*p=*0.047) during the rye bread period (mean intake 208 g ± 38.3 g) compared with wheat bread period (mean intake 170 ± 36.4 g). Plasma glucose did not change. B	B=5	Moderate	Probable protective association between whole-grain consumption and risk of type 2 diabetes
	Inflammatory markers	Intervention studies: *Andersson et al., 2007* (28)*, Sweden.* 3-Day dietary records. Cross-over intervention. No effect of adding 112 g of whole grains to the habitual diet. B *Brownlee et al., 2010* (29)*, UK*. 149-Item FFQ during the preceding 7 day. Intervention 60–120 g whole-grain products daily. No effect. B	B=2	Insufficient	No conclusion on the association between whole-grain consumption and inflammatory markers
	Colorectal cancer	*Larsson et al., 2005* (30), *Swedish women*. 67-Item FFQ at baseline. High consumption of whole grains was associated with lower risk of colon but not rectal cancer. Highest (≥4.5 servings/day) vs. lowest (<1.5 servings/day) RR 0.65 (0.45–0.94) *p=*0.04. Adjusted for age, BMI, education, total energy intake, and intakes of saturated fat, calcium, red meat, fruits, and vegetables. B *Egeberg et al., 2010* (31), *Danish men and women*. 192-Item FFQ. Higher whole-grain product intake (median in total cohort 130 g/day, 5th–95th percentile 42–267 g/day) was associated with lower risk of colon cancer in men. The adjusted IRR was 0.85 (0.77–0.94) per every 50 g/day increment. By quartile categories RR was 0.61 (0.43–0.86) when comparing highest quartile (>160 g/day) with lowest quartile (≤75 g/day). No association among women. Adjusted for BMI, alcohol intake, education, intake of red and processed meat, use of hormone replacement therapy (women only), and leisure time physical activity. B *McCullough et al., 2003* (32)*, US men and women*. 68-Item semi-quantitative FFQ. Multivariate RR: no association when comparing highest quintile (14.5 servings/week) with lowest quintile (0.8 servings/week). Adjusted for age, METS, aspirin, smoking, family history of colorectal cancer, BMI, education, energy, multivitamin use, total calcium, and red meat intake. B *Schatzkin et al., 2007* (33)*, US men and women*. 124-Item FFQ. Whole-grain intake was inversely associated with colorectal cancer risk RR 0.79 (0.70–0.89). The association was stronger with rectal cancer RR 0.64 (0.51–0.81) than colon cancer RR 0.86 (0.75–0.99) comparing highest quintile (1.3 servings/1,000 kcal) with lowest (0.3 servings/1,000 kcal). Adjusted for sex, physical activity, smoking, menopausal hormone therapy in women, intakes of red meat, dietary calcium, and dietary folate, and total energy intake. B	B=4	Low	Suggestive protective association between whole-grain consumption and risk of colorectal cancer
Milk and milk products	CVD/CHD. Stroke (haemorrhage and infarction) Heart failure	*Al-Delaimy et al., 2003* (34)*, US men*. 131-Item FFQ. IHD (fatal and non-fatal). No association between total dairy (RR 1.01, 0.83–1.23) comparing highest quintile with lowest. No separate assessment was performed for specific dairy foods. B *Warensjö et al., 2010* (35)*, Swedish men and women*. 84-Item FFQ. Nested case-control study of incident myocardial infarction with biomarkers of milk fat intake and FFQ data. FFQ: Comparing highest quartile with lowest (>580 vs. 230 g/day): Men: total dairy, OR 1.36 (0.71–2.6) fermented dairy, OR 0.49 (0.26–0.93) cheese, OR 0.60 (0.30–1.20) Women: total dairy, OR 0.45 (0.09–2.32) fermented dairy, OR 0.34 (missing–1.64) cheese, OR 0.38 (0.07–2.22). Biomarker concentrations for milk fat: Lower in cases, especially in women, than in controls and inversely associated with risk markers of metabolic syndrome. B *Bonthuis et al., 2010* (36)*, Australian men and women.* 129-Item FFQ. CVD mortality: no association between total dairy or low-fat dairy, milk, yoghurt, or full-fat cheese and CVD mortality comparing highest tertile (599 g/day) with lowest (174 g/day); however, full-fat dairy associated with CVD mortality RR 0.33 (0.13–0.81). B *Elwood et al., 2004* (37)*, and Elwood et al., 2005* (38)*, British men.* (Note that the men in the 2005 study were part of the 2004 study). FFQ (2004 study) plus 7-d weighted record in 30% of men (2005 study). No significant associations between milk drinking and ischemic heart disease (IHD) or stroke (or combined) (although RR below 1.0 in all) comparing ≥1 pint/day with no milk consumption. Above median milk 187 mL/day compared with below associated with reduced risk of stroke (OR 0.52; 0.27–0.91) IHD OR 0.88 (0.56–1.40). B *Larsson et al., 2009* (39)*, Finnish men, smokers only*. 276-Item FFQ. Stroke: Comparing extreme quintiles (1,296 vs. 287 g/day) of total dairy RR 1.14 (0.99–1.32) for cerebral infarction and 1.32 (0.89–1.94) for intracerebral haemorrhage. No association for separate dairy products except for: whole milk and intracerebral haemorrhage RR 1.41 (1.02–1.96) and cream and cerebral infarction RR 0.81 (0.72–0.92). B	B=6 2 SRs	Insufficient Moderate	No conclusion on the specific direction of the association between dairy/milk consumption and risk of CVD. Altogether (including the SRs), there was no consistent evidence that dairy food consumption in general is associated with increased risk of CVD/CHD (moderate–grade evidence) However, dairy products are a heterogenic group of foods that may have different effects on CVD/CHD. In addition, the studies may have had limited ability to detect the effect of a single food in a mixed diet on complex clinical outcomes
	Type 2 diabetes and biomarkers (insulin resistance,* insulin sensitivity, and glucose tolerance) *IRS=insulin resistance=presence of 2 or more of 4 signs: 1) fasting plasma insulin >20 pU/mL fasting glucose >6.1 mmol/L or medications; 2) obesity >BMI 30 or WHR >0.85 F and 0.90 M; 3) blood pressure >130/80 or medications; and 4) dyslip HDL<0.90 mmol/ or high Tg ≥2.26 mmol/L	Insulin resistance IRS 1 study *Pereira et al., 2002* (40)*, US men and women (18–30 years at baseline).* 700-Item FFQ. Prospective, longitudinal. Dairy consumption inversely associated with incidence of all components of IRS at BMI ≥25 at baseline but not among leaner; multivariable-adjusted OR of developing IRS were 0.28; (0.14–0.58) among overweight in the highest (≥35 times/week. 24/102 individuals) compared with the lowest (<10 times/week. 85/190 individuals) category of dairy consumption. Per each daily increase in dairy consumption a 21% lower odds of IRS: OR. 0.79 (0.70–0.88). Other dietary factors, including macronutrients and micronutrients, did not explain the association. Significant inverse associations observed for most of separate dairy products except butter/cream and yoghurt. B Diabetes incidence 3 Prospective cohort studies: *Choi et al., 2005* (41)*, US men (HPF).* 130-Item FFQ. Dairy consumption (quintile 5 vs. 1), multivariable-adjusted RR 0.77 (0.62–0.95). 9% lower risk per 1 serving/day increase RR=0.91 (0.85–0.97). Stronger for low-fat dairy vs. high-fat dairy *p*-trend 0.001 vs. 0.12. Among individual dairy products, significant inverse association only for skimmed milk. Borderline statistically significant positive associations for whole milk consumption. B *Liu et al., 2006* (42)*, US women (WHS).* 131-Item FFQ. Dairy consumption (quintile 5 vs. 1; >2.9 vs. 0.85 serving/day) associated with RR 0.68 (0.52–0.89) (*p*-trend=0.006) per serving increase RR 0.96 (0.90.1.02). For low-fat dairy RR 0.69 (0.52–0.91) and for high-fat dairy 0.99 (0.82–1.20) comparing quintile 5 with 1. For individual dairy products, inverse association mainly for yoghurt. B *Kirii et al., 2009* (43)*, Japanese men and women*. 147-Item FFQ. Total dairy OR=0.71(0.51–0.98) in women, no association in men, comparing >300 with <50 g/day. No association for milk, cheese, or yoghurt. B Intervention (1 study)	B=7	Low	Suggestive protective association between dairy consumption and type 2 diabetes incidence Total dairy consistently associated with decreased risk in three prospective cohort studies rated B; varying results for specific dairy products but seemed stronger for low-fat than high-fat dairy Less support for the above finding in studies using intermediate biomarkers of type 2 diabetes
		*Barr et al., 2000* (44)*, US men and women*. Adding three 8-ounce servings of skimmed or 1% fluid milk to usual consumption of dairy for 12 weeks: no difference in insulin or HbA1c between intervention group and controls; a slight increase (∼2%) in fasting glucose in intervention group and 1% decrease in control group. B Insulin sensitivity, fasting glucose, and post-load glucose (2 prospective studies)			
		*Ma et al., 2006* (45)*, US men and women*. 114-Item FFQ. Total dairy (average 1 serving/day) not associated with insulin sensitivity. B *Snijder et al., 2008* (46)*, US men and women*. 92-Item FFQ. Dairy consumption (continuous, 0–17 serving/day) at baseline not associated with delta fasting glucose or post-load glucose. Similar for individual dairy products after 6 years. B			
	Inflammatory markers	Intervention study *Wennersberg et al., 2009* (47)*, Finnish, Norwegian, and Swedish men and women*. Middle-aged overweight men and women with traits of the metabolic syndrome. Advised to consume 3–5 servings of dairy/day. No effect on inflammatory markers. A	A=1	Insufficient	No conclusion on the association between dairy consumption and inflammatory markers
	Prostate cancer	*Michaud et al., 2001* (48)*, US men.* 130-Item FFQ. Metastatic prostate cancer risk (stage D and fatal), multivariate model (not adjusting for calcium intake) Dairy >69 g/day vs. <19 g/day; RR 1.43 (0.91–2.3) Skim milk >2 serving/week vs. 0 serving/week; RR 1.25 (0.83–1.9) Whole milk >4 serving/week vs. 0 serving/week; RR 1.25 (0.80–1.9) Cottage cheese ≥2 serving/week vs. 0 serving/week; RR 1.06 (0.75–1.5) Other cheese ≥5 serving/week vs. ≤3 serving/month; RR 1.29 (0.88–1.9) Cream cheese ≥1 serving/week vs. 0 serving/week; RR 1.20 (0.81–1.8) B *Tseng et al., 2005* (49)*, US men.* 105-Item FFQ. Total prostate cancer, multivariate model (not adjusting for calcium intake) Dairy 21 serving/week vs. 5 serving/week; RR 2.2 (1.2–3.9) Total milk 14 serving/week vs. 0.5 serving/week; RR 1.8 (1.1–2.9) Low-fat milk 7 serving/week vs. 0 serving/week; RR 1.5 (1.1–2.2) Whole milk 7 serving/week vs. 0 serving/week; RR 0.8 (0.5–1.3) Cheese 4 pint/week vs. 0.25 serving/week; RR 1.1 (0.6–1.9) Cottage cheese 1 serving/week vs. 0 serving/week; RR 1.2 (0.8–1.8) Cream yes/no; RR 0.9 (0.6–1.3) Yoghurt yes/no; RR 1.0 (0.6–1.9) B *Rohrman et al., 2005* (50)*, US men*. 60-Item FFQ. Total prostate cancer, multivariate model (not adjusting for calcium intake) Dairy >1.9 serving/day vs. <0.9 serving/day; HR 1.08 (0.78–1.54) Cheese >5 serving/week vs. <1 serving/week; HR 1.43 (1.01–2.03) Milk >5 serving/week vs. <1 serving/week; HR 1.26 (0.91–1.74) B *Mitrou et al., 2007* (51)*, Finnish men, smokers only.* 276-Item FFQ. Total prostate cancer, multivariate model (not adjusting for calcium intake*)* Dairy 1,220 g/day vs. 381 g/day; RR 1.26 (1.04–1.51) Total milk 994 g/day vs. 153 g/day; RR 1.08 (0.91–1.30) Whole milk 668 g/day vs. 0 g/day; RR 1.05 (0.86–1.29) Low-fat milk 773 g/day vs. 76 g/day; RR 1.18 (0.97–1.44) Cheese 55 g/day vs. 3 g/day; RR 1.13 (0.95–1.36) Sour milk 423 g/day vs. 0 g/day; RR 1.07 (0.90–1.28) *Similar findings for different stages of prostate cancer* B *Allen et al., 2008* (52)*, men in 8 European countries*. Total prostate cancer, multivariate model (not adjusting for calcium intake) Milk and milk beverages 466 g/day vs. 34 g/day; HR 1.01 (0.89–1.16) Yoghurt 135 g/day vs. 12 g/day; HR 1.17 (1.04–1.31) Cheese 57 g/day vs. 15 g/day; HR 1.04 (0.90–1.20) B *Rodriguez et al., 2003* (53)*, US men.* 68-Item FFQ. Total prostate cancer, multivariate model (not adjusting for calcium intake) Dairy ≥4 serving/day vs. <3 serving/week; RR 1.1 (0.9–1.3) B *Park et al., 2007* (54)*, US men.* 180-Item FFQ. Total prostate cancer, multivariate model (not adjusting for calcium intake) Dairy ≥332 g/day vs. <49 g/day; RR 1.03 (0.92–1.16) Total milk ≥256 g/day vs. <17 g/day; RR 1.07 (0.95–1.19) Low/no-fat milk ≥243 g/day vs. 0 g/day; RR 1.16 (1.04–1.29) Whole milk ≥163 g/day vs. 0 g/day; RR 0.88 (0.77–1.00) Yoghurt ≥40 g/day vs. 0 g/day; RR 0.96 (0.84–1.09) Cheese ≥14 g/day vs. 0 g/day; RR 1.01 (0.91–1.12) After stratification, effects of low/non-fat milk were limited to localised or low-grade tumours. B *Kurahashi et al., 2008* (55)*, Japanese men.* 138-Item FFQ. Total prostate cancer, multivariate model (not adjusting for calcium intake) Dairy 339.8 g/day vs. 12.8 g/day; RR 1.63 (1.14–2.32) Milk 290.5 g/day vs. 2.3 g/day; RR 1.53 (1.07–2.19) Yoghurt 31.5 g/day vs. 1.9 g/day; RR 1.52 (1.10–2.12) Cheese 6.2 g/day vs. 1.9 g/day; RR 1.32 (0.93–1.89) B	B=8	Low	Suggestive increased risk of prostate cancer with increased total dairy consumption based mainly on two prospective cohorts (graded B) with sufficient exposure range and comparatively high intakes in the highest exposure category. In cohort studies with lower intakes and narrower intake ranges, null associations were observed
	Breast cancer	*McCullough et al., 2005* (56)*, US women.* 68-Item FFQ. Total postmenopausal breast cancer. Dairy products ≥2 serving/day vs. <0.5 serving/day; RR 0.81 (0.69–0.95) reduced risk of postmenopausal breast cancer. The association was slightly stronger in women with oestrogen receptor-positive tumours, comparing highest to lowest intake. B *Pala et al., 2008* (57)*, women in 8 European countries.* Pre- and postmenopausal breast cancer, multivariate model (not adjusting for calcium intake) Total milk 439 g/day vs. 0 g/day; HR 1.05 (0.97–1.14) Whole milk 150 g/day vs. 0 g/day; HR 1.06 (0.97–1.15) Semiskim 293 g/day vs. 0 g/day; HR 1.05 (0.97–1.12) Skim 210 g/day vs. 0 g/day; HR 0.93 (0.87–1.01) Cheese 82 g/day vs. 6 g/day; HR 0.97 (0.89–1.06) No consistent association with total breast cancer risk. B *Shin et al., 2002* (58)*, US women.* 61–130-Item FFQ updated*.* Premenopausal diet for premenopausal breast cancer, multivariate model (not adjusting for calcium intake) Total dairy foods >3 serving/day vs. ≤1 serving/day; RR 0.73 (0.55–0.86) Skim/low-fat milk >1 serving/day vs. never; RR 0.72 (0.56–0.91) No association was found between pre/postmenopausal intake of dairy products and postmenopausal breast cancer. Among premenopausal women, high intake of low-fat dairy foods, especially skim/low-fat milk, was associated with reduced risk of breast cancer. B	B=3	Insufficient	No conclusion on the association between dairy consumption and risk of breast cancer
	Bone health	*Feskanich, 2003* (59)*, US women*. FFQ. No association between milk consumption (5 categories ranging from <1/week to >1.5/day) and risk of hip fractures (*p*-trend=0.21 for continuous intakes). B	B=1	Insufficient	No conclusion on the association between dairy consumption and bone health
Meat	CVD/CHD	*He et al., 2003* (60)*, US men.* Semiquantitative FFQ (3 times). Outcome: ischemic and haemorrhagic stroke Ischemic stroke: Red meat (in frequency) ≥1/d vs. <1/wk; RR 0.97 (0.60–1.55) Haemorrhagic stroke: Red meat (in frequency) ≥1/day vs. <1/week; RR 1.58 (0.55–4.55) No significant association between red meat and ischemic/haemorrhagic stroke. B *Steffen et al., 2007* (61)*, US men and women.* 66-Item semiquantitative FFQ (interview administered at baseline and 6 years later). Outcome: venous thromboembolism (VTE) Red and processed meat >1.5 serving/day vs. <0.5 serving/day; HR 2.01 (1.15–3.53) Red and processed meat intake was significantly associated with risk of VTE. B *Nettleton et al., 2008* (21)*, US men and women*. 66-Item semiquantitative FFQ (interview administered at baseline and 6 years later) Outcome: Incident heart failure (HF) RR for HF incidence according to intake of red or processed meat (RR representing expected changes in risk of HF per 1 serving/day difference in food group consumption): RR 1.27 (1.18–1.37) when adjusted only for energy intake. Red or processed meat was not significantly associated with HF risk after multivariable adjustment; RR 1.07 (0.97–1.17)*.* B *Sinha et al., 2009* (62)*, US men and women.* 124-Item FFQ. Outcome: CVD mortality In men (5th vs. 1st quintile): Red meat: 68.1 g/1,000 kcal vs. 9.3 g/1,000 kcal, HR 1.27 (1.20–1.35); processed meat: 19.4 g/1,000 kcal vs. 5.1 g/1,000 kcal, HR 1.09 (1.03–1.15). In women (5th vs. 1st quintile): Red meat: 65.9 g/1,000 kcal vs. 9.1 g/1,000 kcal, HR 1.50 (1.37–1.65); processed meat 16.0 g/1,000 kcal vs. 3.8 g/1,000 kcal, HR 1.38 (1.26–1.51) Increased CVD mortality for both men and women with increased intake of red and processed meat. B	B=4	Insufficient	No conclusion on the association between red meat/processed meat and CVD/CHD The end-point diversity of these 4 studies contributes to the unclear conclusion
	Inflammatory markers	No studies		Insufficient	No conclusion on the association between red or processed meat consumption and inflammatory markers
	Iron status	*Szymlek-Gay et al., 2009* (66)*, New Zealand*. 20-month RCT among 12–20-month-old toddlers; red meat group, milk group, and control group. Participants in the red meat group were encouraged to eat ≥2 portions of red meat/day (56 g). However, compliance was low and mean number of meat dishes consumed was only 0.7/day. No intervention effect on prevalence of suboptimal iron status. In the red meat group there was no change in serum ferritin during the intervention, but at week 20, serum ferritin was significantly higher in the meat than the control group (*p=*0.033). B	B=1	Insufficient	No conclusion on the association between red meat consumption and iron status
	Colorectal cancer	*WCRF/AICR:* World Cancer Research Fund/American Institute for Cancer Research. Continuous Update Project Report Summary. Food, Nutrition, Physical Activity, and the Prevention of Colorectal Cancer (2011) (6). Nine of 12 studies of colorectal cancer demonstrated increased risk with higher intake of red meat. Meta-analysis: 17% increased risk of colorectal cancer per 100 g/day intake. Indication of increased risk of colon and rectal cancer but non-significant. Ten of 13 studies of colorectal cancer demonstrated increased risk with higher intake of processed meat. Meta-analysis: 18% increased risk of colorectal cancer and 24% increased risk of colon cancer per 50 g/day intake. Indication of increased risk of rectal cancer but non-significant.	…	…	Red and processed meat are convincingly associated with increased risk of colorectal cancer

* Based on apparently healthy population.

### Potatoes

With respect to the three outcomes (CVD/CHD, type 2 diabetes, and inflammatory markers) examined in relation to potato consumption, only one paper met the quality criteria (for diabetes, graded B) (Table 5). In the US Nurses’ Health Study, consumption of potatoes (mainly deep-fried, baked, or mashed) was associated with increased risk of diabetes, which persisted in obese but not in lean women after stratification. The one study of CVD was graded C and not included. No articles were retrieved in the updated search. Therefore, no conclusion could be drawn regarding the associations between potato consumption and CVD/CHD, type 2 diabetes, or inflammatory markers.

### Berries

Two studies – graded B – were included examining the association between berry consumption and CVD/CHD incidence or mortality in the general population. Both of these observational studies were carried out in the United States, which makes their implications for Nordic countries questionable. Nevertheless, no conclusion could be drawn because of the inadequate number of studies, which in addition produced mixed results.

No studies of the association between berry consumption and type 2 diabetes were identified. Five intervention studies examining the effect of berry consumption on inflammatory markers produced mixed results. Therefore, no conclusion could be drawn even for this endpoint.

### Whole grains

Out of the seven identified studies, all conducted in the United States, exploring the association between whole-grain consumption and CVD/CHD incidence or mortality, six met the quality criteria (graded B). These studies consistently indicated that whole-grain consumption was associated with decreased risk of CVD/CHD incidence or mortality; the evidence grade was moderate for total CVD, indicating a probable protective association. It should be noted, however, that the evidence was insufficient for specific outcomes such as stroke or heart failure.

Five observational studies and one intervention study (examining the effects of rye bread on plasma glucose and insulin response) of type 2 diabetes and intermediate biomarkers of type 2 diabetes met the quality criteria (all graded B). In the observational studies, the highest or second highest exposure quintile/quartile was consistently associated with lower risk of incident type 2 diabetes. In the intervention study, whole-grain consumption increased the insulin response but had no effect on plasma glucose. The evidence of the effect of whole grains on diabetes was considered moderate in strength, leading to the conclusion that higher whole-grain intake probably reduces the risk of type 2 diabetes. Most of the studies were carried out in the United States, but a Finnish study produced similar results. Notably, the whole-grain intake was much higher among the participants in Finland, the lowest quartile median intake (79 g/day) in Finland being higher than the highest quintile mean intake (45.6 g/day) found in US studies.

The effect of whole-grain consumption on inflammatory markers was examined in two good-quality studies (graded B), both indicating null associations. Therefore, because of insufficient evidence, no conclusion could be drawn regarding the effect of whole grains on inflammatory markers.

Four out of five studies of the association between whole-grain consumption and colorectal cancer incidence or mortality in the general population met the quality criteria (all graded B). These large cohort studies were conducted in Sweden, Denmark, and the United States. In three of four studies, significant associations were found between whole-grain consumption and reduced colorectal cancer risk, whereas one study obtained null results. The exposure variables were estimated in very different ways across studies, potentially contributing to the mixed results. Nevertheless, the evidence grade was considered low, indicating a suggestive protective association.

### Milk and milk products

Six outcomes were considered in relation to dairy consumption (Table 5). Six studies of CVD/CHD met the quality criteria and were graded B; two SRs were also identified. No significant associations were observed between total dairy consumption and any of the CVD endpoints. A separate analysis of specific dairy products obtained mixed results. The two SRs concluded that the evidence was either insufficient (63) or that there was no consistent evidence that dairy food was associated with higher risks of CHD (64). Based on this SR, the evidence was rated insufficient regarding any specific dairy product or outcome, so no conclusion was drawn regarding the direction of the association between dairy or milk consumption and CVD risk. Nevertheless, it was concluded (including in the identified SRs) that no consistent evidence indicates that dairy food consumption is associated with an increased risk of CVD/CHD (moderate-grade evidence). The additional articles identified in the updated search in February 2012 supported the above conclusion.

Seven studies of type 2 diabetes and of the intermediate biomarkers of diabetes met the quality criteria, all graded B. Total dairy consumption was consistently associated with decreased risk of diabetes in four prospective cohort studies, three studies of diabetes incidence, and one study of insulin resistance (from the United States and Japan). The results varied for specific dairy products, but seemed stronger for low-fat than high-fat dairy. Less support was observed in two studies of insulin sensitivity and blood glucose. In the one randomised intervention study identified, adding three servings of fluid milk per day to the diet did not affect the concentration of HbA1c; the evidence grade was low, and the conclusion was that there is a suggestive protective association between dairy consumption and type 2 diabetes. The updated search found two additional studies indicating inverse associations between dairy consumption and type 2 diabetes incidence and one intervention study obtaining null results for an intermediate biomarker, altogether supporting the above conclusion.

No conclusion could be drawn regarding milk and milk product consumption and inflammatory markers and bone health, respectively, because of insufficient evidence; the articles retrieved in the updated search supported this result.

Eight studies of milk and milk product consumption and prostate cancer met the quality criteria, all graded B. Increased risk was observed in some studies with comparatively higher exposure levels and a sufficient exposure gradient. Increased risk was observed in a Finnish study, in which the results were similar for different stages and grades of cancer. Increased risk was also observed in a US study. Similarly, a Japanese study observed an increased risk although the intake was lower. However, no significant association was observed in a US study at relatively high intakes, that is, dairy ≥4 servings/day vs. <3 servings/week. The definition of dairy exposure, however, varied between the studies (e.g. some included ice cream while others did not). In a US study, an increased risk was observed for low-fat milk only, but after stratification by tumour grade, the significant associations with low- and nonfat-milk were limited to localised or low-grade tumours. Although the results were mixed, an association with increased risk could not be excluded, leading to the conclusion – supported by low-grade evidence – of a suggestive increased risk of (total) prostate cancer with dairy or milk consumption. Evidence is insufficient to draw conclusions regarding specific dairy products.

### Red and processed meat

Four studies of CVD/CHD met the quality criteria and were graded B. In two of these studies with varied CVD endpoints, increasing red meat (including processed meat) intake was significantly associated with increased risk, whereas no significant association was observed in two studies. These four studies indicate that red meat (including processed meat) may increase the risk of CVD endpoints; however, the insufficient number of studies, their endpoint diversity, and their insufficiently strong evidence meant that a firm conclusion could not be drawn. A systematic review and meta-analysis (65) of papers from the 1990s until 2009 concluded that consumption of processed meat, but not red meat, is associated with higher incidence of CHD (and diabetes mellitus). However, another SR (63) concluded that the evidence was insufficient.

The WCRF update (6) identified 12, 10, and 7 studies of the association between red meat consumption and the risk of colorectal, colon, and rectal cancer, respectively. Nine of 12 colorectal cancer studies demonstrated increased risks with higher red meat intake. Meta-analysis demonstrated a 17% increased risk of colorectal cancer per 100 g/day of red meat consumption. An indication of increased risk of colon and rectal cancer was seen, but was not statistically significant. The WCRF update identified 13, 11, and 10 studies of the association between processed meat consumption and the risk of colorectal, colon, and rectal cancer, respectively. Ten of 13 colorectal cancer studies demonstrated increased risks with higher processed meat intake. Meta-analysis demonstrated an 18% increased risk of colorectal cancer and a 24% increased risk of colon cancer per 50 g/day consumption. There was a non-significant indication of an increased risk of rectal cancer. WCRF concluded that red meat and processed meat consumption is a convincing cause of colorectal cancer. No additional studies or SRs contradicting these conclusions were found in the updated search.

We identified no relevant studies of the association between red meat or processed meat and inflammatory markers.

Five studies of iron status were identified, only one of which met the quality criteria (graded B). The RCT by Szymlek-Gay et al. demonstrated that increased intake of red meat among 12 to 20-month-old toddlers improved the iron status (66). Based on this SR, the evidence was rated insufficient and no conclusion was drawn. No additional studies or SRs contradicting these conclusions were found in the updated search.

## Discussion

This SR focused on original research articles treating five food groups common in the Nordic diet, that is, potatoes, berries, whole grains, milk and milk products, and red and processed meat, and their associations with health outcomes. A sufficient number of studies meeting the predefined eligibility and study quality criteria were required to make judgements regarding the scientific evidence concerning these foods’ influence on health and wellbeing. Because of limited numbers of studies, conclusions could only be drawn for whole grains, milk and milk products, and red and processed meat regarding certain research questions. This SR found moderate-grade evidence for a probable protective association between high whole-grain intake and the risks of CVD and type 2 diabetes, whereas low-grade evidence indicated a suggestive protective association between whole-grain consumption and colorectal cancer risk. The evidence grade was also low for high dairy intake, indicating both a suggestive protective effect against type 2 diabetes and a suggestive increased risk of prostate cancer. However, this SR concluded, based on moderate-grade evidence, that dairy products are not associated with increased risk of CVD. The evidence for positive associations between red and processed meat intakes and increased risk of CVD was considered insufficient, so no conclusion could be drawn, although some studies reported a clearly increased risk. Based on the WCRF/AICR review, we conclude that red and processed meat consumption is a convincing cause of colorectal cancer. Results for all other research questions were limited and non-conclusive.

The major strength of this SR is its predefined set of methods: all reviewers used the same predefined eligibility criteria to identify and select articles, and followed the same procedure to extract information from each article and to evaluate study quality (3). To date, very few published reviews have used such strict and objective criteria to judge scientific evidence in nutrition research. The procedure implemented by the current review project is adopted from procedures developed by the Agency for Healthcare Research and Quality, (AHRQ), US Department of Health and Human Services (4, 5), and WCRF/AICR (2).

A clear limitation of the SR is that very few articles meeting both the eligibility and quality criteria were found, partly because the literature search included only studies published after 1999. This lack of qualifying articles restricted the possibility of drawing conclusions. Even though the number of studies focusing on food consumption rather than intakes of specific nutrients has increased in recent years, more articles potentially meeting both the eligibility and quality criteria would likely have been found if a longer time period had been covered. The SR was restricted to considering original research articles. Systematic literature reviews and meta-analyses were included only if these applied eligibility criteria and quality assessments to the reviewed studies similar to those applied here. With regard to the quality grading, very few of the eligible original articles were graded A, most being classified as B (or C). Moreover, because of the three-grade quality system, grade B was assigned to studies of a wide range of quality levels. It is important to note that, because strict eligibility and quality criteria were used, some studies may have been downgraded or excluded because information was lacking regarding certain study design features. Thus, the apparent lack of high-quality studies may to some extent depend on the limited information provided by authors. The undue influence of measurement error and confounders is always a danger in nutritional epidemiology; however, the quality assessment performed likely minimised such influences.

The SR identified very few studies of potatoes and berries. In addition, because most of those studies were conducted in the United States and very few were set in the Nordic countries, the applicability of the results is limited. Potatoes are traditionally consumed boiled (not deep-fried) in the Nordic countries, so both the quality and quantity of exposures may differ between populations and studies. Similar differences likely exist for berry consumption between Nordic and other populations; for example, the effect of sea buckthorn berries, which are not a common food item in the Nordic diet, was examined in two out of five intervention studies.

Whole grains were the only food group for which the evidence was rated moderate (i.e. for CVD and type 2 diabetes). Nevertheless, the definitions of whole-grain consumption and the amounts consumed varied across studies and populations. This may have hampered the drawing of conclusions for some of the outcomes, such as colorectal cancer (low–grade evidence).

Dairy is a heterogenic group of foods that may have varied and opposing health effects. A need was identified for more detailed hypotheses regarding milk and milk products, and for studies focusing on specific dairy products. A report from the Danish National Food Institute (67), reviewing cohort and case control studies of dairy consumption, considered high consumption of milk and milk products to reduce the risk of stroke, type 2 diabetes, and colorectal cancer, and probably to increase the risk of prostate cancer. However, the Danish report concluded that milk and milk product consumption did not affect CHD risk and did not increase the risk of breast cancer.

The endpoint diversity in the reviewed studies of the association between red and processed meat intake and CVD contributed to the conclusion of insufficient evidence. An SR and meta-analysis (65) including papers from the 1990s until 2009 concluded that consumption of processed meat, but not red meat, is associated with higher incidence of CHD, whereas another SR (63) graded the evidence as insufficient for a causal relationship between meat consumption (not specified) and risk of CHD.

Two recent meta-analyses and one comprehensive review by Alexander et al. (68–70) of processed meat and red meat consumption and colorectal cancer found summary relative risks similar to those found by the WCRF/AICR, but concluded that the available evidence was insufficient to support a clear positive association between processed meat and colorectal cancer. However, the review did not meet the criteria for SRs and was partially funded by meat producer organisations.

The WCRF/AICR advocates increased consumption of plant food at the expense of animal food products, red and processed meat in particular (2, 6). Since it is well known that meat consumption is an important contributor to iron intake, there is concern that limiting red and processed meat consumption may put certain population groups at higher risk of mineral (i.e. iron) deficiency. In this SR, the only relevant study included found that increased intake of red meat among 12 to 20-month-old toddlers improved their iron status. More studies are needed to be able to draw firmer conclusions. In a recent Nordic project (71), the overall nutritional consequences of lowering the daily consumption of meat from current levels to the level suggested by the WCRF/AICR (i.e. under 500 g cooked red meat/week, very little if any processed), with specific emphasis on reducing intake of processed meat, was assessed. Tetens et al. demonstrated that the current intake level (mean values of groups stratified by age and gender) of red meat is not far from the WCRF-recommended level at the individual level according to national dietary survey data from the Nordic countries (71). When modelling a scenario of the total average diet at the group level where the intake of processed meat was reduced to 0, the re-estimated nutrient content indicated little effect on iron intake. However, the study did not include toddlers, and it was noted that it must be considered that the bioavailability of dietary iron may decrease with decreased meat intake.

Until now, few published intervention studies have examined the health impact of specific Nordic diets (72) or of diets planned according to NNR guidelines using ordinary Nordic foods (73, 74). These studies have consistently demonstrated important health benefits for intermediate biomarkers of CVD. As descriptive studies have revealed the detrimental qualities of foods included in current diets in northern compared with southern European countries, with low vegetable and fruit consumption and higher intakes of sweets, pre-prepared food products, and animal foods in the north (75, 76), the need to identify the health-contributing factors of the traditional Nordic diet is even more pressing.

To conclude, there was not enough evidence to draw any conclusions regarding the health impact of potatoes and berries based on this SR. There was probable evidence (moderate-grade) that whole-grain intake was protectively associated with type 2 diabetes and CVD. This SR also found suggestive evidence (low-grade) that dairy consumption was associated with decreased risk of type 2 diabetes and with increased risk of prostate cancer. There were too few studies to draw any conclusions regarding red meat intake and CVD risk. In addition, the WCRF/AICR concludes that red meat and processed meat is a convincing cause of colorectal cancer. This SR revealed the need for more high-quality studies of the specific features of the Nordic diet.
